# Evaluation of a novel oral mucosa in vitro implantation model for analysis of molecular interactions with dental abutment surfaces

**DOI:** 10.1111/cid.12750

**Published:** 2019-03-12

**Authors:** Sanne Roffel, Gang Wu, Ivana Nedeljkovic, Michael Meyer, Tojo Razafiarison, Susan Gibbs

**Affiliations:** ^1^ Department of Oral Cell Biology, Academic Centre for Dentistry Amsterdam (ACTA) University of Amsterdam and Vrije Universiteit Amsterdam Amsterdam The Netherlands; ^2^ Department of Oral Implantology and Prosthetic Dentistry, Academic Centre for Dentistry Amsterdam (ACTA) University of Amsterdam and Vrije Universiteit Amsterdam Amsterdam The Netherlands; ^3^ Department of Dental Material Sciences, Academic Centre for Dentistry Amsterdam (ACTA) University of Amsterdam and Vrije Universiteit Amsterdam Amsterdam The Netherlands; ^4^ Nobel Biocare Services AG Kloten Switzerland; ^5^ Department of Molecular Cell Biology and Immunology, Amsterdam Infection and Immunity Institute Amsterdam UMC, Vrije Universiteit Amsterdam Amsterdam The Netherlands

**Keywords:** abutment, dental implant, in vitro, junctional epithelium, model, organotypic, reconstructed human gingiva, reconstructed oral mucosa, soft tissue attachment

## Abstract

**Background:**

Abutment surfaces are being designed to promote gingival soft tissue attachment and integration. This forms a seal around prosthetics and consequently ensures long‐term implant survival. New scalable and reproducible models are necessary to evaluate and quantify the performance of these surfaces.

**Purpose:**

To evaluate a novel implantation model by histomorphometric and immunohistochemical characterization of the interactions between human oral gingival tissue and titanium abutments with either novel anodized or conventional machined surface.

**Materials and Methods:**

Abutments were inserted into an organotypic reconstructed human gingiva (RHG) model consisting of differentiated gingival epithelium cells on a fibroblast populated lamina propria hydrogel following a tissue punch. Epithelial attachment, down‐growth along the abutment surface, and phenotype were assessed via histomorphology, scanning electron microscopy, and immunohistochemistry 10 days after implantation.

**Results:**

The down‐growing epithelium transitioned from a gingival margin to a sulcular and junctional epithelium. The sulcus depth and junctional epithelial length were similar to previously reported pre‐clinical and clinical lengths. A collagen IV/laminin 5 basement membrane formed between the epithelium and the underlying connective tissue. The RHG expanded in thickness approximately 2‐fold at the abutment surface. The model allowed the evaluation of protein expression of adhering soft tissue cells for both tested abutments.

**Conclusions:**

The RHG model is the first in vitro 3D model to enable the assessment of not only human epithelial tissue attachment to dental abutments but also the expression of protein markers involved in soft tissue attachment and integration. The two abutments showed no noticeable difference in epithelial attachment.

## 1 INTRODUCTION

Modifications to implant surfaces are being investigated to improve the clinical performance of dental implants. In addition to modifications to the surface of the implant body, which are aimed at promoting osseointegration, abutment surfaces are being modified to support soft tissue attachment, maintenance of soft tissue health, and reduction in bacterial adhesion. The attachment of the soft tissue to the tooth or implant/abutment surface is necessary to form a biological seal that protects the underlying connective tissue and bone from microorganisms. As has been previously described, pathogenic microbial colonization can lead to periimplantitis and bone resorption culminating in dental implant failure.^1^, ^2^

The soft tissue in which the dental implant (or tooth) is embedded is called the gingiva. The gingiva consists of the epithelium, which forms the outermost barrier between the individual and the environment, and the vascularized connective tissue. The epithelium lining the outer surface of the gingiva is adapted to its biological function and can be recognized by its distinct histology and the expression of specific keratins. The free gingival margin is the visible part of the gum, which is covered on the luminal side by a keratinized epithelium expressing keratin 4 but not keratin 19.^3^ Further interior from the free gingival margin epithelium is the oral sulcular epithelium, which lines the gingival sulcus. This sulcus is the space between the gingiva and the surface of the tooth, which contains the crevicular fluid. Continuing on from the sulcular epithelium is the nonkeratinized junctional epithelium, which expresses keratin 19 but not keratin 4, and which is the first epithelium that is directly attached to the tooth. The junctional epithelium therefore plays an extremely important role in forming a tight biological seal against microbial colonization of the underlying tissues. In a healthy situation, the junctional epithelium is approximately 2 mm in height on average. It tapers off in the apical direction, ranging from 15 to 30 cell layers coronally to 1 to 3 cell layers apically. The junctional epithelium is connected to the underlying lamina propria via the external basal lamina, which contains collagen IV and laminin 5, and to the tooth via the internal basal lamina, in which collagen IV is absent. The epithelial attachment to both basal lamina is via hemidesmosomes.^4^, ^5^ Proliferating keratinocytes, which express Ki67, are found adjacent to the external basal lamina, where they serve as a reservoir of cells to replenish differentiated cells, which are shed off at the apical end of the sulcular and junctional epithelium. Prior research has focused on optimal osseointegration and connective tissue attachment to implant materials and abutments. Very little is known, however, about the optimal function and the attachment of the junctional epithelium to these materials.^1^, ^2^, ^6^, ^7^

Not surprisingly, surface chemistry not only appears to play a role in bone integration but also in soft tissue integration.^8^ Dental abutments are made of primarily titanium material, due to its great mechanical properties and proven biocompatibility.^9^ A titanium dioxide layer with a thickness of approximately 5 nm, which forms naturally on the titanium surface when exposed to air or water, has been shown to improve corrosion resistance and biocompatibility.^10^ Therefore, various titanium dioxide modification techniques have emerged to further enhance the wound healing process.^11^ Among the techniques used, titanium surface anodization has proven beneficial in promoting soft tissue attachment to dental abutments in studies ranging from in vitro cellular experiments to clinical trials.^12^, ^13^, ^14^, ^15^, ^16^

There are few physiologically relevant models for studying soft tissue attachment to an abutment surface. Current models rely heavily on animal experiments often including dogs and pigs.^17^, ^18^, ^19^ Such animal models should be kept to a minimum according to the European Directive 2010/63/EU, which is based on the principle of the Three R's, to Replace, Reduce, and Refine the use of animals used for scientific purposes. In addition, such models are often limited in terms of scalability and ability to conduct extensive cellular analyses and findings may not be representative of human outcomes.^20^ In vitro alternatives have the advantage of lower variability and easier access to the site under investigation (ie, no manipulation in the constraints of an animal's oral cavity is necessary), and such models allow for the quantification of the strength of the attachment between the cells and the abutment using pull‐out force measurements. Simple in vitro 2D‐culture methods have been used extensively.^21^, ^22^ These 2D models do not resemble the human organotypic physiology, however, and are not suitable for testing final products, which have different geometries and surfaces. Due to these significant limitations, there is an unmet need for the development of human organotypic and physiologically relevant gingiva models to assess soft tissue attachment to new abutment surfaces at a molecular level. Ideally, such models would also allow for the functional evaluation of the strength of the seal. In the future, such models may even allow for the quantification of the strength of the attachment between the cells and the abutment by pull out force measurements.

The aim of the present study was to evaluate a novel in vitro organotypic 3D model that allows for both histomorphologic characterization of the soft tissue attachment to dental abutments and protein marker expression analysis.^20^ As previously described, our 3D organotypic reconstructed human gingiva (RHG) consists of a fully differentiated gingiva epithelium (telomerase reverse transcriptase [TERT] immortalized keratinocytes) on a lamina propria (TERT immortalized fibroblast populated collagen hydrogel). The advantage of using TERT immortalized cells is that production protocols can be standardized to produce large numbers of RHG, thus avoiding the complicated logistics involved in obtaining small, highly variable, and often infected biopsies for culture. This TERT RHG has been extensively characterized and compared to the primary cell counterpart and native gingiva biopsies. The gingival epithelium has similar K10, K13, involucrin, and loricrin expression to native gingiva.^23^ The model has been further validated with respect to inflammatory cytokine release after chemical exposure and introduction of full thickness wounds.^23^, ^24^, ^25^, ^26^ The TERT‐RHG is therefore a promising tool to develop further into a novel in vitro implantation model. To assess the soft tissue attachment using this model, two abutment surface technologies with identical macrodesigns were selected: a novel anodized surface and an unmodified surface. Limitations of this model were also assessed including the impact of a lack of underlying bone, difficulty in separating the abutment from the culture, and the influence of transformed cells.

## 2 MATERIALS AND METHODS

### 2.1 Abutment details

In this study, two abutments types made of titanium alloy (Ti6AI4V) were used. The first abutment type (surface 1) was a Nobel Biocare On1 NP of 2.5 mm collar height with a machined surface (Nobel Biocare AB, Gothenburg, Sweden). The second abutment type (surface 2) was a Nobel Biocare On1 NP of 2.5 mm collar height with a novel anodized surface (Nobel Biocare). Both abutment types were sterilized and sealed in blister packages. Also in this supplement, Milleret et al. report that both abutment types present the same surface roughness but with different surface chemistry (manuscript accepted CID‐18‐335).

### 2.2 RHG culture and abutment placement

Immortalized human gingiva keratinocyte (KC‐TERT, OKG4/bmi1/TERT, Rheinwald Laboratory, Boston, Massachusetts)^27^, ^28^ and fibroblast cell lines (Fib‐TERT, T0026, ABM, Richmond, British Columbia, Canada) were used to construct the RHG as previously described.^23^ The RHG were cultured at the air‐liquid interface in a cell culture incubator (37°C, 95% humidity) in culture medium (DMEM/Ham's F12 (3/1); Gibco, Grand Island, New York) supplemented with 1% Fetal Clone III (RHG, Logan, Utah), 1% penicillin‐streptomycin (Gibco), 0.1 μM insulin (Sigma‐Aldrich, St. Louis, Missouri), 2 μM hydrocortisone (Sigma‐Aldrich), 1 μM isoproterenol (Sigma‐Aldrich), 10 μM carnitine (Sigma‐Aldrich), 10 mM L‐serine (Sigma‐Aldrich), 0.4 mM L‐ascorbic acid (Sigma‐Aldrich), and 2 ng/mL epidermal growth factor (Sigma‐Aldrich) for 10 days. During this time, a differentiated epithelium formed on a fibroblast‐populated collagen hydrogel.

After 10 days of air‐exposed culture, abutments were inserted into the RHG as follows: a 3‐mm diameter tissue punch (Kai Medical, Solingen, Germany) and tweezers were used to remove a full thickness biopsy from the center of each RHG. Abutments were carefully removed from sterile packaging using a titanium‐coated tweezers and gently placed into the 3‐mm diameter holes so that the abutment surface was in close contact with the RHG. The RHG with abutments was then placed carefully into the culture incubator and evaluated at a single time point to quantify the soft tissue attachment at 10 days after insertion. Culture medium was exchanged every 3 to 4 days. Three independent experiments were performed, each with an intraexperiment duplicate.

### 2.3 Histomorphometric analysis

Each RHG with the attached abutment was rinsed in saline and then chemically fixed in buffered 10% formaldehyde solution (Merck KGaA, Darmstadt, Germany) for 1 day at 4°C, followed by rinsing in tap water, dehydrating in ethanol, and embedding in methylmethacrylate.^29^ Using a microtome (Leica SP1600, Leica Biosystems, Wetzlar, Germany), the tissue blocks were cut through the longitudinal axis of the implants into 250‐μm‐thick slices (3‐4 total, 500 μm apart) according to a systematic random sampling protocol.^30^ All slices were then glued to Plexiglas specimen holders and ground down to a final thickness of 80 to 100 μm. The slices were then surface‐polished and surface‐stained with McNeal's Tetrachrome, basic Fuchsine, and Toluidine blue.^31^ The microscopic sections were visualized and recorded with a Nikon Eclipse 80i microscope. Epithelial down‐growth along the abutment surface was determined from photographs using NIS‐Elements AR 2.10 imaging software (Nikon Instruments Europe B.V., Amsterdam, The Netherlands).

### 2.4 Scanning electron microscopy

Abutments were carefully removed from the RHG with tweezers to visualize epithelial keratinocyte attachment to the abutment surface. Abutments with epithelial layers were fixed in 1% glutaraldehyde (Merck KGaA, Darmstadt, Germany) and 4% formaldehyde (Merck KGaA) in 0.1 M sodium cacodylate (Merck KGaA) buffer for 2 to 3 days and postfixed in 1% osmium tetroxide for 2 hours. This procedure was followed by dehydration in a series of ascending ethanol concentrations at 50%, 70%, 90%, and 100% for 15 minutes each with two changes of each solution. Thereafter, the samples were sputter‐coated with gold using an Edwards Sputter Coater S150B (Edwards, Burgess Hill, England) and examined in a Zeiss EVO LS‐15 scanning electron microscope (Zeiss, Oberkochen, Germany).

### 2.5 Histology and immunohistochemistry

Abutments were carefully removed from the RHG with tweezers; care was taken not to damage the epithelium attached to the collagen hydrogel. The samples were fixed in 4% formaldehyde and processed for conventional paraffin embedment. Paraffin sections of 5 μm thickness were cut, deparaffinized, and rehydrated in preparation for morphological analysis (hematoxylin and eosin staining) or immunohistochemical analysis of keratin 4 (6B10, Thermo Fisher Scientific, Naarden, The Netherlands), keratin 19 (RCK108, Dako, Glostrup, Denmark), Ki67 (MIB‐1, Dako, Glostrup, Denmark), laminin 5 (Novus Biologicals, Abingdon, United Kingdom), and collagen IV (CIV22, Monosan, Uden, The Netherlands). Antigen retrieval on paraffin sections was performed to detect keratin 4, keratin 19, Ki67, laminin 5, and collagen IV. Ki67 sections were immersed in 0.01 M sodium citrate buffer (pH 6.0) for 30 minutes at 100°C, followed by slow cooling to room temperature and washing in PBS. For keratin 19, laminin 5, and collagen IV, a protease digestion step with pepsin (Dako, Glostrup, Denmark) was performed for 8 minutes at 37°C. For keratin 4, a Tris/EDTA pH 9.0 antigen retrieval was performed for 10 minutes at 100°C followed by slowly cooling to room temperature. After fixation and antigen retrieval, sections were washed in PBS and incubated with secondary antibody for 1 hour at room temperature followed by incubation with Poly‐HRP‐Anti Ms/Rb IgG complex (BrightVision+ System, Immunologic, Amsterdam, The Netherlands). All sections were washed in PBS and counterstained with hematoxylin. The microscopic sections were visualized and recorded with a Nikon Eclipse 80i microscope using NIS‐Elements AR 2.10 imaging software (Nikon Instruments Europe B.V.).

### 2.6 Data analysis

Three independent experiments were performed, each with an intraexperiment replicate.

**Figure 1:**
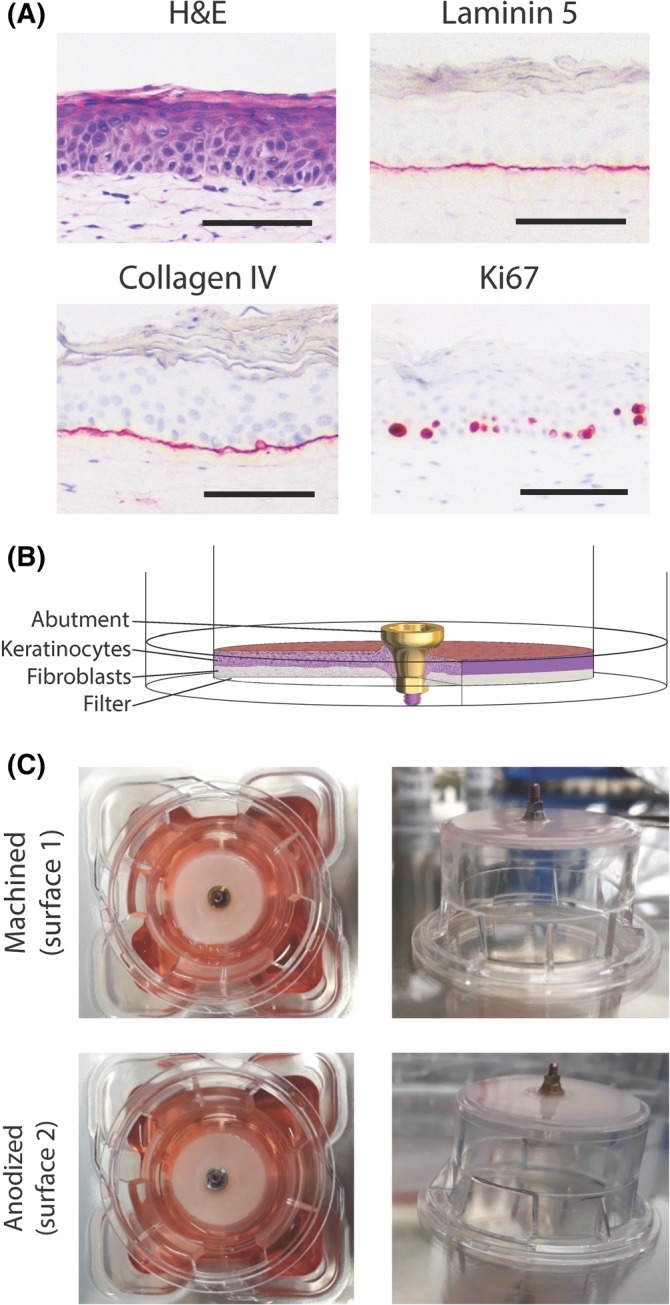
The reconstructed human gingiva (RHG) implantation model. A, Immunohistochemical analysis of paraffin embedded tissue sections is shown. Magnification bar = 100 μm. B, A scheme showing the experimental design. C, A macroscopic view of the implants placed into the RHG. The RHG is shown at the time of harvesting, which was after 10 days of culture at the air‐liquid interface. The transwell diameter = 2.5 cm. Representative results obtained from 6 images and 3 independent experiments are shown; see Materials and Methods, section Data Analysis for further information

For histomorphometric measurements, tissue sections, as indicated in Figure 1, were analyzed as follows: for each independent experiment, values obtained from the intraexperiment replicates including measurement of the left and right side of the implant (total of four measurements) were first averaged, and then this value was used to determine the average of the three independent experiments. Differences were determined using one‐way ANOVA followed by Dunnett's uncorrected multiple comparisons test using GraphPad Prism version 7.02 for Windows, GraphPad Software, La Jolla, California. Differences were considered significant when *P < 0.05.

Immunohistochemical and histomorphometric images are the representative of 12 images derived from the tissue sections of the 3 independent experiments each with an intraexperiment replicate RHG, which also had epithelium growing adjacent to the left and right side of the implant.

For scanning electron microscopy, a single RHG from each of the three independent experiments was analyzed.

## 3 RESULTS

### 3.1 Epithelial down‐growth parallel to the abutment surfaces

The RHG model used in this study is shown in Figure 1. It consists of a differentiated stratified epithelium (7‐9 viable cell layers) on a fibroblast‐populated collagen hydrogel. Proliferating Ki67‐positive keratinocytes are present in the basal layer. A collagen IV/laminin V positive basal lamina is observed at the interface of the epithelium and hydrogel. After 10 days of culture at the air‐liquid interface, the RHG was attached to both abutment surfaces to such an extent that the abutments remained in place when the cultures were inverted (Figure 1C).

**Figure 2:**
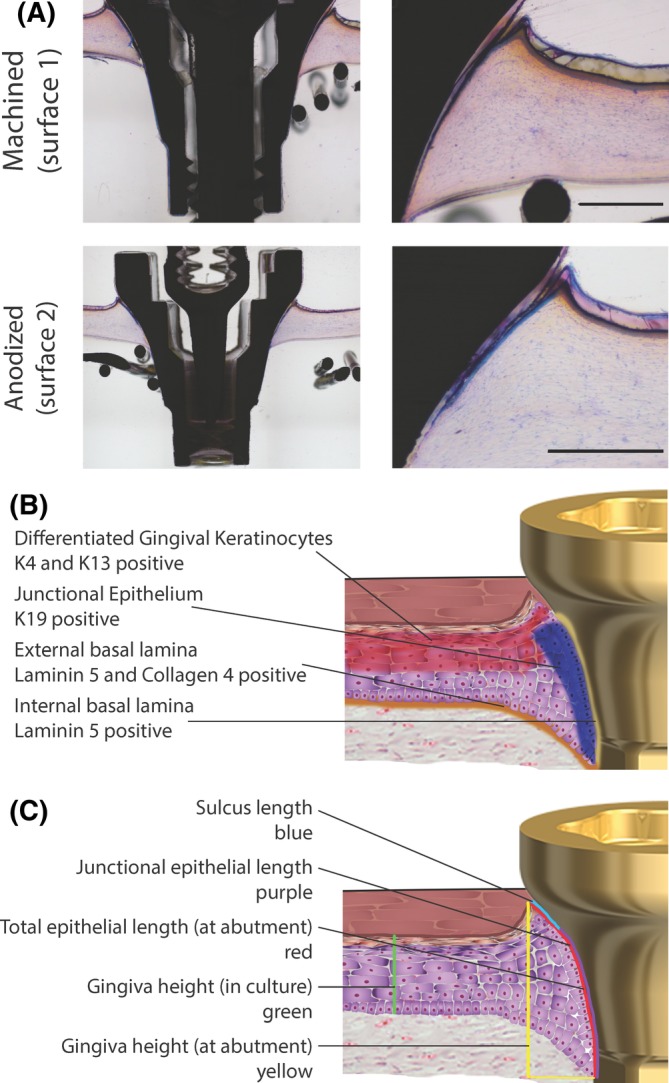
Histomorphometric analysis shows reconstructed human gingiva (RHG) attached to machined (surface 1) and anodized (surface 2) surfaces. A, Tissue sections 80‐100 μm thick were surface‐polished and surface‐stained with McNeal's Tetrachrome, basic Fuchsine, and Toluidine blue. Representative results obtained from 12 images and 3 independent experiments are shown; see Materials and Methods, section Data Analysis for further information. Scale bar = 500 μm. B, A schematic representation of the RHG implantation model, which indicates tissues of interest and their protein expression. C, A schematic representation of the RHG implantation model with a visualization of parameters that were measured histomorphometrically

Histomorphometric analysis was used to assess the epithelial down‐growth along the different abutment surfaces (Figure 2). Notably, an area of no attachment, which resembled the sulcus, was observed immediately adjacent to the upper surface of the RHG on both abutment surfaces (Figure 2A). The epithelium was further observed to grow downwards parallel to the abutment surfaces, tapering off from 7 to 9 living cell layers at the upper coronal surface to 1 to 2 cell layers at the lower apical surface, thus resembling the junctional epithelium. Because the in vitro down‐growing epithelium resembled both the sulcular and junctional epithelium observed in human and animal studies, similar measurement criteria were used to histomorphologically assess the RHG (Figure 2A,C; Table 1).^19^, ^32^ Notably, for both abutment surfaces, the RHG expanded in thickness approximately 2‐fold at the abutment surface, and the epithelium (soft tissue) in contact with the abutment surface was 86% to 88% of the total length (1561 and 1508 μm for surfaces 1 and 2, respectively) (Table 1).

**Table 1:** Histomorphometric measurements

Parameter	Surface 1	Surface 2
Sulcus depth (μm; SD)	143 ± 42	148 ± 55
Junctional epithelium length (μm; JE)	1070 ± 82	963 ± 56
Gingiva Height (μm; GH)	1268 ± 32	1205 ± 45
Culture Height CH (μm)	585 ± 7	552 ± 10
Total length (μm; TL) SD + JE + NC)	1561 ± 32	1508 ± 66
Hydrogel Length not in contact with JE (μm; NC)	182 ± 56	209 ± 78
Soft tissue in contact with surface (%) ([TL ‐ NC] / TL x 100)	88 ± 3.6	86 ± 4.8
Gingiva expansion at abutment surface (GH / CH)	2.17 ± 0.03	2.21 ± 0.14

Histomorphometric measurements were performed as shown in the schematic drawing in Figure 2. Histomorphometric analysis was based on 12 images for each surface. For each of the 3 independent experiments, values from intra‐experiment replicates, including the internal left and right images derived from a single tissue section were first averaged and then the average of the 3 independent experiments is shown ± SEM. No significant differences were observed between surface 1 and surface 2.

### 3.2 Epithelium attachment to abutment surfaces

**Figure 3:**
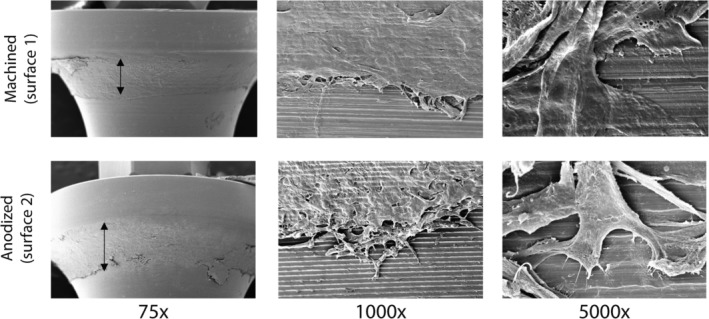
Scanning electron microscopy showing epithelial cell attachment to the abutment surfaces. Left: double headed: The arrow indicates the width of the attached epithelium. Middle: An example of the migrating epithelial front. Right: An example of keratinocyte attachment to the abutment surface. Numbers indicate the fold magnification. Representative results obtained from 3 independent experiments are shown; see Materials and Methods, section Data Analysis for further information

Because histomorphometric analyses showed epithelial down‐growth parallel to the surface of both abutments, we next investigated the extent of epithelial attachment to the different surfaces using SEM, which is a technique that has been previously used to assess soft tissue attachment.^33^ The abutments were gently dissected from the RHG without the use of enzymatic digestion to ensure that the epithelial keratinocytes remained strongly attached to the surfaces after removal of the RHG collagen hydrogel (Figure 3). An epithelial cell layer was observed (75x magnification) to cover the surface region of both abutments, corresponding to the junctional epithelial length (Table 1; Figure 2C). Higher magnification (1000x) showed a confluent epithelial sheet in close contact with each abutment surface and keratinocytes extending from the migrating epithelial front onto the abutment surfaces. The highest magnification (5000x) clearly showed individual keratinocytes spread and attached to abutment surfaces via filopodia extensions.

### 3.3 Epithelium adjacent to abutments develops sulcular and junctional epithelial characteristics

**Figure 4:**
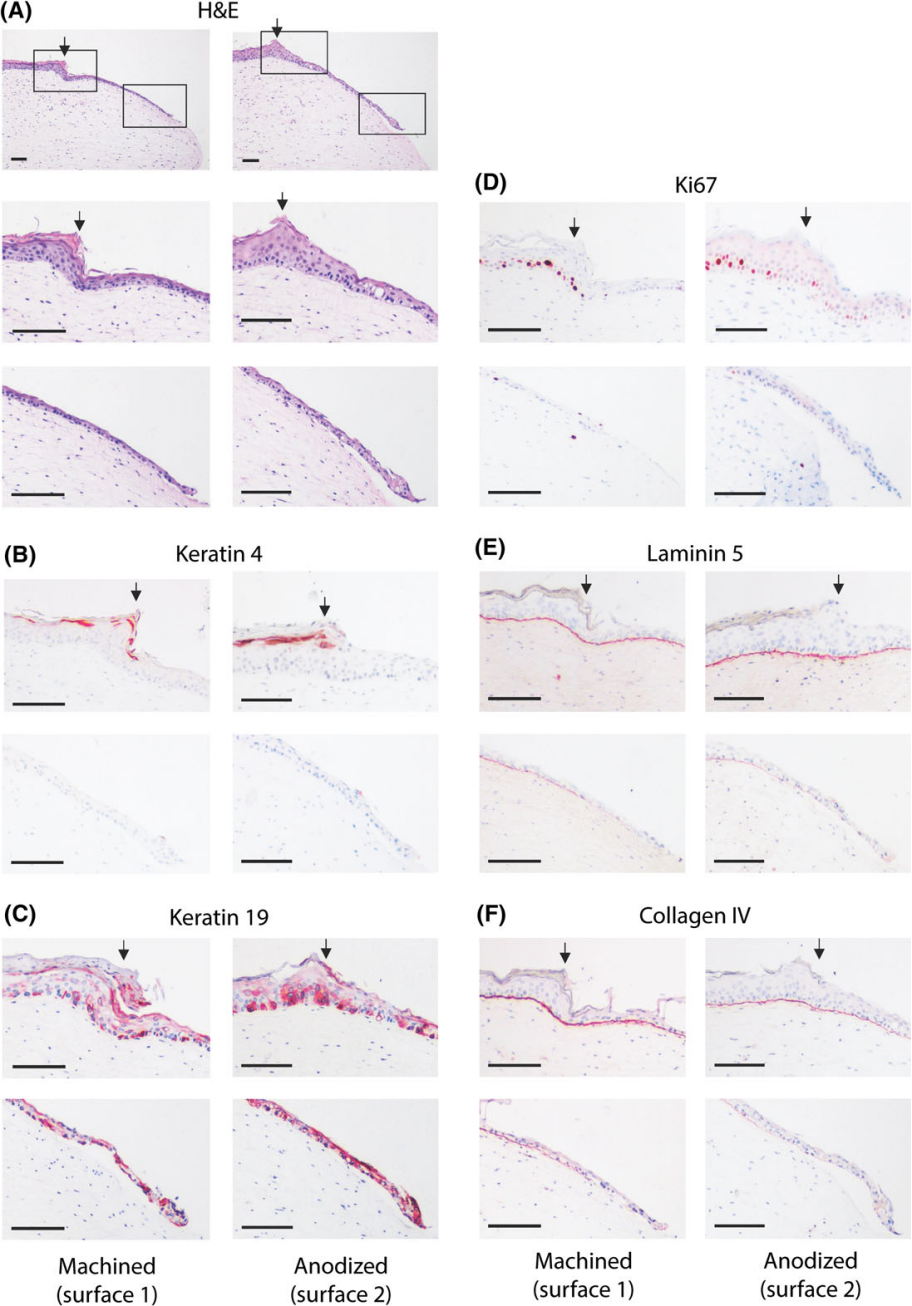
Immunohistochemical analysis of down‐growing reconstructed human gingiva (RHG) epithelium adjacent to the abutment surface. A, Tissue sections (5 μm) were stained with hematoxylin and eosin (H&E) to visualize the histology. A stratified and cornified gingiva epithelium is observed in the RHG in the area that was not in contact with the abutment surface (to the left of the arrow). Down‐growing epithelium can be observed to the right of the arrow. The solid box left represents the upper panels following staining, and shows the transition of the epithelium phenotype as it comes into contact with the abutment surface. The solid box right represents the lower panels following staining, and shows the migrating front of the junctional epithelium adjacent to the abutment surface. B‐F, Immunohistochemistry using antibodies directed against epithelial biomarkers (keratin 4 [K4] or keratin 19 [K19]), proliferation marker Ki67, or basement membrane proteins collagen IV and laminin 5 (red immune‐staining). Scale bar = 100 μm. Representative results obtained from 12 images and 3 independent experiments are shown; see Materials and Methods, section Data Analysis for further information

**Figure 5:**
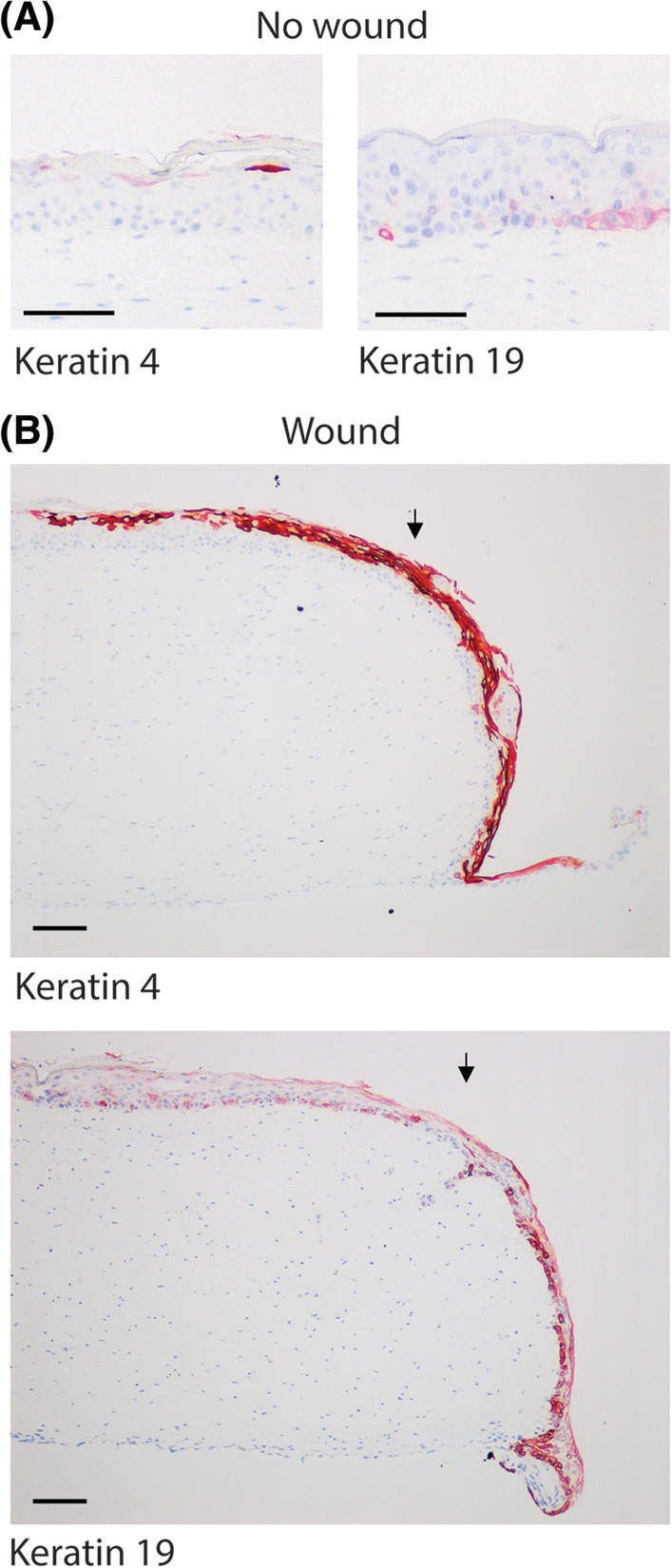
Immunohistochemical analysis of control reconstructed human gingiva (RHG) without wound and RHG with wound but without abutment. Tissue sections (5 μm) were stained with antibodies directed against epithelial biomarkers keratin 4 or keratin 19 (red immune‐staining). Heamatoxlyin staining shows cell nuclei (blue). Scale bar = 100 μm. Representative results obtained from 12 images and 3 independent experiments are shown; see Materials and Methods, section Data Analysis for further information

Next, an immunohistochemical analysis of the RHG epithelium and basal lamina region in the vicinity of the abutment surfaces was performed. Figure 4 shows the tissue sections of the RHG after the abutments were carefully removed, leaving the epithelium attached to the fibroblast‐populated collagen hydrogel. A stratified and cornified gingival epithelium was observed in the area of the RHG that was not in contact with the abutment surface (Figure 4). Keratin 4, a gingival epithelial protein, was expressed in the upper epithelial cell layers, whereas junctional epithelial keratin 19 was only intermittently expressed in the undifferentiated basal cell layers (Figure 4). For both abutments, the epithelium became less differentiated as it expanded downwards along the abutment surface and tapered off until it was only 1 to 2 cell layers thick, which was consistent with the histomorphometric analysis (Figure 2). The lower epithelial layers no longer expressed keratin 4 but instead strongly expressed the junctional epithelial keratin 19 in all keratinocytes. As controls for the study, unwounded and wounded RHG without implants were studied (Figure 5). In line with the epithelium not in contact with the abutment surfaces, K4 was expressed in differentiated cells in the uppermost layer, and K19 was expressed only in sporadic cells within the basal layer of unwounded RHG. In contrast, in the down‐growing regenerating epithelium of wounded RHG (no implant), K4 was very strongly expressed in all cells, and K19 was strongly expressed only in the basal cells of the down‐growing epithelium. This protein expression is therefore not typical of junctional epithelium or the RHG epithelium growing adjacent to the implant surfaces, which was K4 negative, K19 high (Figure 4). For both surfaces, proliferating Ki67‐stained keratinocytes were observed in the gingiva epithelium but not in the actively down‐growing epithelium adjacent to the abutment surfaces (Figure 4). In addition, lamina propria basement membrane proteins collagen IV and laminin 5 were observed for both surfaces and formed a distinct line of expression between the down‐growing epithelium and hydrogel. Neither protein was observed on the outer epithelium side adjacent to the abutment surface. Although it was not possible to perform statistical analysis of the immunohistochemical staining, the results were extremely consistent within and between the three independent experiments.

## 4 DISCUSSION

In this study, we assessed the suitability of the RHG model for the evaluation of soft tissue attachment to different abutment surfaces at both the histomorphometric and immunohistochemical level. We focused on soft tissue structures, namely the sulcular and junctional epithelium and their associated protein expression patterns. Notably, the down‐growing epithelium adjacent to both abutment surfaces adapted its phenotype to resemble a gingival margin, sulcular, and junctional epithelium and expressed the associated physiological proteins on both abutment surfaces. Therefore, this study shows for the first time that an organotypic culture model can exhibit the features representative of human oral mucosa epithelium attachment to an implant surface.

Because the in vitro down‐growing epithelium resembled the sulcular and junctional epithelium, the same measurement criteria that are used in human and animal studies were used to assess the RHG in this study^19^, ^32^ and to compare with human clinical data.^5^, ^34^ It is important to consider, however, that the dynamics of wound healing may be different in this model compared to preclinical and clinical studies. For the end point of 10 days, we observed sulcus depths of 143 ± 42 μm (anodized surface) and 148 ± 55 μm (machined surface), which is smaller depth than has been observed in human clinical studies (1.2 mm). The in vitro junctional epithelium tapered off from 7 to 9 living cell layers at the upper coronal surface to 1 to 2 cell layers at the lower apical surface, however, which is consistent with human data. In humans, the junctional epithelium is estimated to be 1.4 to 3.3 mm, which is slightly longer than the range observed in our study.

The histological observations further support the use of the RHG model. Differences in keratin 4 and 19 expression were observed in the gingival margin (K4^high^, K19^low^), sulcular (K4^high^, K19^low^), and junctional (K4^negative^, K19^high^) epithelium, closely resembling the expression pattern found in clinical analyses.^3^ The in vitro RHG also notably expanded in thickness approximately 2‐fold at the abutment surface, which is another physiologically relevant characteristic of the model.^5^ The smaller sulcus depth and junctional epithelial values obtained in the in vitro RHG are possibly explained by the limiting height of the hydrogel (approximately 1 mm) and the short duration of the experiment (10 days). In future studies, a thicker hydrogel and a longer culture period may be able to more closely mimic the length of the native gingiva, which is in the range of 3 mm.^35^

Collagen IV and laminin 5 were both expressed at the interface between the collagen hydrogel and sulcular epithelium, as well as the junctional epithelium, indicating that an external basement membrane was forming due to crosstalk between keratinocytes and fibroblasts in the hydrogel.^36^ Both of these basement membrane proteins were absent at the interface between the abutment surface and the down‐growing epithelium. In in vivo rat studies, the internal basement membrane, which forms at the interface of the tooth and the epithelium, expresses laminin 5 but not collagen IV.^5^ Because the junctional epithelium that forms around implants originates from epithelial cells of the mucosa rather than from reduced enamel epithelium^5^ (as is the case for junctional epithelium adjacent to teeth),^5^ it cannot be determined from our current findings whether (1) cells expressing these proteins were torn away with the removed implant; (2) the RHG model cannot develop an internal basement membrane; or (3) collagen IV as well as laminin 5 are not deposited at the implant surface in humans. Future applications of the RHG model will be able to investigate this question once harvesting methods have been further optimized to prevent tearing of the epithelium.

The current RHG model is a wound healing model in which the active migration of epithelial cells along the abutment surface occurs simultaneously with lower epithelial cell proliferation.^37^ By contrast, in vivo junctional epithelium exhibits a high rate of keratinocyte proliferation along its length adjacent to the external basement membrane. This limitation of the RHG model may possibly be prevented in the future by using longer cultivation times, which may result in homeostasis with less migration and more proliferation, and thus may be more representative of healed tissue around the abutment. The trade‐off is that longer cultivation time will result in more epithelial down‐growth due to the lack of underlying bone, which is expected to yield extreme nonphysiological junctional epithelial lengths. An evaluation of the time course of growth in a future study is needed to generate measurements that are more relevant to soft tissue attachment, migration, and junctional epithelium on dental implant surfaces. Once such limitations are addressed, however, the RHG implantation model is expected to be a valuable tool for conducting preclinical efficacy studies in a scalable, reproducible, and cost‐effective manner and will allow for the possibility of more detailed analysis, such as protein expression and pull‐out force measurements, which are not possible using traditional models. Another limitation associated with the lack of underlying bone is the absence of a reference point frequently used for measurement in clinical and preclinical studies.

Chai et al. also developed an RHG‑implant model, which in a number of ways is similar to our model.^20^, ^38^, ^39^ They investigated epithelial attachment of titanium discs inserted into RHG constructed from primary human oral mucosa keratinocytes and fibroblasts seeded on the top of human acellular dermis. A limitation of this model is the scalability and sourcing of primary cells for constructing RHG. In‐line with our study, histomorphometric analysis and SEM showed epithelial down‐growth and attachment, and additionally transition electron microscopy showed some hemidesmosome‐like structures. They also investigated the quality of biological seal with the aid of tritiated water. However, unlike in our study, they did not perform extensive characterization (eg, immunohistochemistry) of the epithelium forming adjacent to the titanium disks in order to determine whether it represented junctional epithelium nor did they perform an analysis correlating to the measurement criteria that are used in human and animal studies. Furthermore, Chai et al. used titanium discs, which may be expected to perform differently to abutments, which have a different weight, conical form and threads, and so forth. Most interesting, this group has further developed their RHG model to include an underlying bone‐like structure consisting of rat osteosarcoma cells seeded into a hydroxyapatite/tri‐calcium phosphate scaffold to mimic alveolar bone.^40^

In conclusion, the RHG model is the first organotypic in vitro model that enables the assessment of soft tissue epithelial attachment to dental abutments using the same parameters that have been defined for use in clinical and animal efficacy studies. Our results show that both abutment surfaces equally supported epithelial attachment and keratinocyte spreading at the defined time point of 10 days after insertion. Future studies should aim to examine these parameters at different time points to compare the performance of the surfaces during the healing process. Furthermore, assessing the performance of different surfaces in challenged situations, such as repeated removal of abutments or growth and/or migration inhibition may also more closely mimic clinically relevant situations. Another factor that will need to be addressed in future studies is the effect of the weight and macrostructure of the implants on the parameters; complex normalization techniques will be required to enable the model to be used to evaluate implants from different sources. Finally, it would be desirable to test the attachment strength of the RHG to the abutment using pullout measurements, which would allow for the quantification of a functional parameter not measured traditionally and therefore would be a clear advantage of this model compared to traditional preclinical models.

## CONFLICT OF INTERESTS

This study was financed in part by Nobel Biocare Services AG. MM and TR are the employees of Nobel Biocare. All other authors have no conflicts of interest to declare.
